# Dynamic Non-Rigid Objects Reconstruction with a Single RGB-D Sensor

**DOI:** 10.3390/s18030886

**Published:** 2018-03-16

**Authors:** Sen Wang, Xinxin Zuo, Chao Du, Runxiao Wang, Jiangbin Zheng, Ruigang Yang

**Affiliations:** 1 Northwestern Polytechnical University, Xi’an 710072, China; wangsen1312@gmail.com (S.W.); wangrx@nwpu.edu.cn (R.W.); zhengjb@nwpu.edu.cn (J.Z.); 2 University of Kentucky, Lexington, KY 40506, USA; chao.du@uky.edu; 3 Baidu Inc., Beijing 100085, China

**Keywords:** 3D reconstruction, RGB-D sensor, non-rigid reconstruction

## Abstract

This paper deals with the 3D reconstruction problem for dynamic non-rigid objects with a single RGB-D sensor. It is a challenging task as we consider the almost inevitable accumulation error issue in some previous sequential fusion methods and also the possible failure of surface tracking in a long sequence. Therefore, we propose a global non-rigid registration framework and tackle the drifting problem via an explicit loop closure. Our novel scheme starts with a fusion step to get multiple partial scans from the input sequence, followed by a pairwise non-rigid registration and loop detection step to obtain correspondences between neighboring partial pieces and those pieces that form a loop. Then, we perform a global registration procedure to align all those pieces together into a consistent canonical space as guided by those matches that we have established. Finally, our proposed model-update step helps fixing potential misalignments that still exist after the global registration. Both geometric and appearance constraints are enforced during our alignment; therefore, we are able to get the recovered model with accurate geometry as well as high fidelity color maps for the mesh. Experiments on both synthetic and various real datasets have demonstrated the capability of our approach to reconstruct complete and watertight deformable objects.

## 1. Introduction

3D scanning or modeling is a challenging task that has been extensively studied for decades due to its vast applications in 3D printing, measurement, gaming, etc. The availability of low cost commodity depth sensors, such as Microsoft Kinect, has made the static scene modeling substantially easier than ever. Many scanning systems has been proposed exploiting rigid alignment algorithms to deal with static objects or scenes, e.g., indoor modeling [1,2,3]. However, the limitation to static or rigid scenarios prevents broader applications where the scene or the subject might move or deform in a non-rigid way. Considering the much higher dimensionality and complexity of the deformation space than purely rigid motion, non-rigid objects modeling in dynamic scenario is much more difficult than the static case. In this paper, we will tackle the 3D modeling problem of deformable objects with a single RGB-D sensor.

There have been ways to accommodate deformable objects using color images to track the motion and then reconstruct the 3D shapes [4,5,6]. There are quite a lot of previous works [5,7] that have been done on multi-view stereo that utilize multiple color cameras to reconstruct the 3D model by exploiting the photo consistency constraints together with some smoothness regularizations. Vlasic [6] propose deforming a pre-scanned model under the constraints of multiple images. However, those methods suffer from the ambiguities of appearance matching and also the color variation caused by the illumination effects and view changes. More recently, researchers have taken advantages of the depth sensors while adopting the multi-view setup [8,9,10]. By now, the most recent state-of-art method using multiple cameras has been proposed in [11], which has exploited the temporal information to generate consistent models in time space. Those systems with multiple depth sensors have demonstrated impressive results on dynamic objects modeling. However, they are not portable and often require very precise calibration between those multiple sensors. This makes the 3D modeling with a single depth sensor more attractive.

Many follow-up systems [12,13] have specifically looked at scanning humans where the user rotates in front of the Kinect while maintaining a roughly rigid pose. There are others that incorporate human template (e.g., SCAPE [14] or skeleton [15]) as the prior information and deform the template to align with the input. In this paper, we will focus on reconstruction of deformable objects without any template and also with no need to keep any certain pose. As compared to some previous dynamic fusion works [16,17,18] that suffer from the error accumulation problem, the main contribution of this paper is that we address this drifting problem by enforcing the loop closure constraints explicitly. We have also exploited the captured color images to resolve the ambiguity that exists in non-rigid surface alignment with purely geometric information.

We propose a global non-rigid registration and fusion optimization framework to deal with the error accumulation problem utilizing both geometric and appearance information. In more detail, first, we decompose the input sequence into continuous segments and fuse the frames in each segment to get a partial model (fragment). Those neighboring fragments can be aligned pairwise under our non-rigid registration approach. Next, we detect the loop between those fragments and establish correspondences between fragments that form a loop. Correspondences from the loop closure constraints together with those achieved from pairwise registration procedure are fed into a global non-rigid registration framework. We will get a fused model after the global registration. Then, this fused model will be taken as a proxy model, which is used to facilitate better alignment between those fragments so that the proxy model gets updated to be confronted with all of those fragments. Finally, we are able to generate a watertight 3D model with consistent and clear color maps.

**Figure 1:**
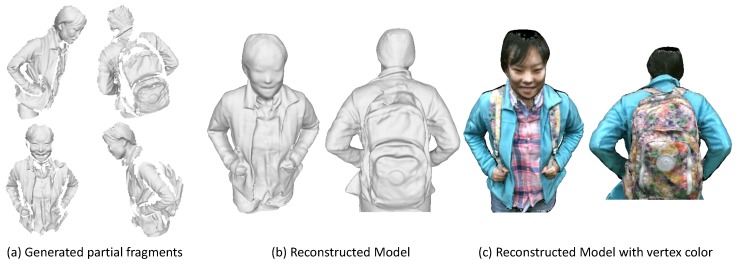
The reconstructed 3D model with our approach. (**a**) some sampled partial scans or fragments; (**b**) the reconstructed model using our approach; (**c**) is our recovered mesh model shown with color maps.

We have evaluated the proposed approach on both a synthetic dataset and several real datasets of deformable objects captured with an RGB-D sensor. As shown in Figure 1, the experimental results demonstrate that our approach is capable of generating high quality and complete 3D models with high fidelity of recovered color maps.

## 2. Related Works

In this paper, we focus on the 3D modeling of non-rigid deformable objects. It is an even harder problem as compared with the rigid object reconstruction problem considering the more complex non-rigid motion. Researchers have proposed various ways to address this problem.

We review some related approaches that use only a single depth sensor for the non-rigid object reconstruction. There are some papers that specify their modeling targets as some pre-scanned models or human body. The pre-scanned model makes the occlusion problem easier to handle as the overall shape is already available. For example, in Ref. [19], the template is pre-scanned and built up first and then got deformed to fit the input acquired from a depth sensor. Later on, Guo [20] improves the surface tracking performance by incorporating both L0 and L2 regularizations. Refs. [21,22] focus on 3D modeling of human body and exploits the prior knowledge in the form of SCAPE model. Therefore, instead of tracking the deformation of all those vertices on the surface, they solve the coefficients of a SCAPE model. Those prior information or human template are enforced to reduce the search space of the overall solution. Another way of reducing the complexity of the non-rigid reconstruction problem and making it more tractable is to set some restrictions on the movement of the target. For example, Li [12] and Zhu [23] have presented the system that asks the user to rotate in front of the sensor while keeping a certain static pose. In addition, the user is also assumed to perform a loop closure explicitly at the end of the sequence. This is restrictive and it may not be easy to hold the same pose during rotation.

Recently, as an extension to the KinectFusion system, Newcombe et al. [16] has proposed the dynamic fusion approach that takes non-rigid motion into account with a non-rigid warp field updated with respect to every frame. The current input gets fused to the canonical model under the current warp field. Later on, Ref. [17] incorporates sparse feature matches into the framework to resolve the ambiguities in alignment. Guo [24] takes advantage of the dense color information to improve the robustness of surface tracking. They have also decomposed the lighting effect from the image to eliminate the color variation affected by the environment lighting. Yu et al. [25] enforce the skeleton constraints in the typical fusion pipeline to get better performance on both surface fusion and skeleton tracking. Those methods allow the user to move more freely. However, they haven’t dealt with the loop closure problem, which makes them not suitable for complete model recovery considering the almost inevitable drifting problem as the sequence proceeds. This issue has been addressed in paper [26] with the proposed non-rigid bundle adjustment method. They have obtained some pleasant results, but the bundle adjustment could be quite expensive and time-consuming due to the number of unknowns and also the search space being quite large. In addition, the recovered color maps of the 3D model is blurry as they haven’t incorporated any color information. In this paper, we will deal with the loop closure problem in a more efficient way. Finally, a complete 3D model together with clear color maps will get reconstructed.

## 3. Pipeline

**Figure 2:**
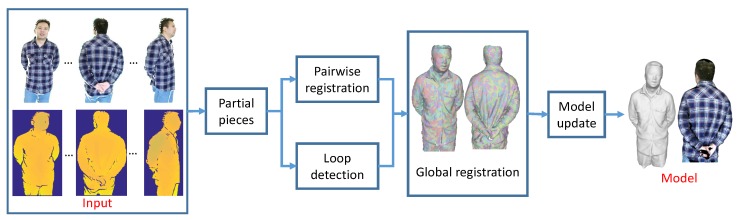
The pipeline of our method.

We illustrate the overall pipeline of our method in Figure 2.

First, given an RGB-D sequence as input, instead of trying to fuse them continuously altogether, we partition it into several segments. We are able to reconstruct a locally precise surface fragment or partial scan from each such segment [17]. Then, those partial scans will be aligned with their neighboring pieces under our pairwise non-rigid registration procedure. Next, we apply a globally non-rigid registration procedure to align those pieces altogether. This is accomplished first by our loop detection process. When the loop is detected, we try to align these pieces that form a loop. Correspondences established between these pieces are enforced in the global registration process. After that, we will get a fused proxy model by merging all the pieces. Finally, in our model update step, we use the proxy model as a starting point to refine the correspondences so as to achieve better alignments afterwards. During the registration process, we have exploited both geometric and color information to register partial pieces and align them altogether. Therefore, we arrive at a complete high quality 3D model together with consistent color maps eventually.

## 4. Our Approach

In this section, we will describe our framework step by step with partial pieces generation, pairwise non-rigid registration, loop closure detection, global registration and finally the model-update.

### 4.1. Partial Pieces Generation and Pairwise Non-Rigid Registration

#### 4.1.1. Partial Pieces Generation

**Figure 3:**
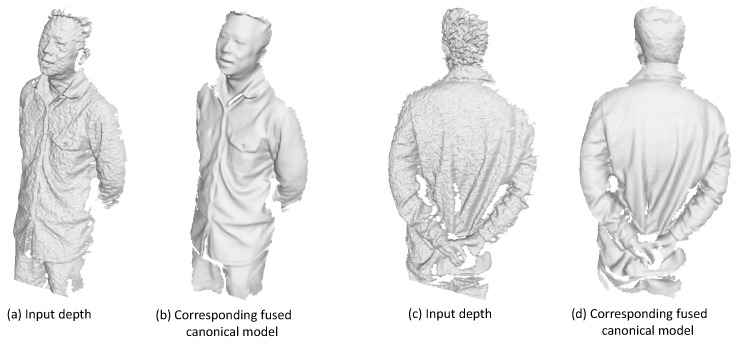
Some sampled partial pieces generated from the fusion procedure. (**a**,**c**) are some input frames; (**b**,**d**) are corresponding partial scans.

We begin our approach by dividing the input RGB-D sequence into *N* continuous segments and extract high quality but only partial scans of the model from each segment exploiting the free form dynamic fusion method [17]. In this method, the working space is defined by a volume with each voxel containing the signed distance value with respect to the surface. A rotation and translation vector is also associated with each voxel to describe its motion or deformation from the canonical space. The surface is represented by these signed distance functions. Typically, the first frame of each segment is taken as the canonical frame. For every input frame, the motion field will be calculated and optimized to get the deformed surface to be confronted with the input depth map. Afterwards, the input depth data can be fused into the canonical model under the guidance of the motion field. The signed distance value in each voxel gets updated and the voxel color is also fused. As the sequence proceeds, the canonical model will get enhanced with some geometric details and occlusion parts revealed. Some examples of reconstructed partial scans are demonstrated in Figure 3. More details can be referred in [17]. We denote those reconstructed canonical meshes as $M_{1}∼M_{N}$. In the meantime, as we keep tracking the motion of each voxel, we will also get the deformed models corresponding to the last frame of every segment. We denote those deformed surfaces as $S_{1}$–$S_{N}$. Those deformed models will be used to guide the pairwise registration in the next section.

Thereafter, our goal is to fuse all those partial pieces $M_{1}$–$M_{N}$ to generate a complete 3D model. We will achieve this in three steps: pairwise registration, global non-rigid registration with loop closure and finally model update/refinement process. Next, we will illustrate each of these steps in detail in the following sections.

#### 4.1.2. Pairwise Non-Rigid Registration

In this section, we describe our approach to register those canonical models non-rigidly and pairwise with their neighboring frames. That is, we try to compute the dense deformation field from $M_{k-1}$ to $M_{k}$ so that $M_{k-1}$ is aligned with its following neighoring piece $M_{k}$. The reason for this pairwise registration is that we want to find the reliable matches between neighboring pieces that can be enforced during the global non-rigid registration process. We accomplish this by exploiting the Embedded Deformation Model [27] to parametrize the deformation of mesh $M_{k-1}$. The key point is that we do not need to specify and calculate the motion parameters for each vertex. Instead, a set of graph nodes $g_{1}$–$g_{l}$ are uniformly sampled throughout the mesh and, for each node $g_{i}$, it has an affine transformation specified by a 3×3 matrix $A_{i}$ and a 3×1 translation vector $t_{i}$. For each vertex v, it gets deformed as driven by its *K* nearest graph nodes with a set of weights $\omega_{j}\left(v\right)$:

(1) $$\phi \left(v\right)=\sum_{j=1}^{K}\omega_{j}\left(v\right)\left[A_{j}\left(v-g_{j}\right)+g_{j}+t_{j}\right].$$

In our case, we take $M_{k-1}$ as the source mesh and $M_{k}$ as the target mesh. We randomly sample a set of graph nodes ($g_{1}$–$g_{l}$) on the mesh $M_{k-1}$ to build up the embedded graph. In order to find the optimal alignment from mesh $M_{k-1}$ to $M_{k}$, deformation parameters $A_{1}$–$A_{l}$ (denoted as A) and $t_{1}$–$t_{l}$ (denoted as T) are optimized by minimizing the following objective function:

(2) $$E\left(A,T\right)=\alpha_{r}E_{r}\left(A\right)+\alpha_{s}E_{s}\left(A,T\right)+\alpha_{g}E_{g}\left(A,T\right)+\alpha_{c}E_{c}\left(A,T\right),$$

where $\alpha_{r}$, $\alpha_{s}$, $\alpha_{g}$, $\alpha_{c}$ are the weights for each term. Next, we explain each of those constraints in detail.

First, the term $E_{r}\left(A\right)$ serves as the as-rigid-as-possible term that specifies that the affine transformations ($A_{1}∼A_{l}$) should try to keep properties of a rotation matrix so as to prevent arbitrary surface distortion:

(3) $$E_{r}\left(A\right)=\sum_{i=1}^{l}\left|\right|A_{i}^{T}A_{i}-I{\left|\right|}_{F}^{2}.$$

Next, the smoothness constraints $E_{s}\left(A,T\right)$ assure the similarity of the local transformations between connected graph nodes. This ensures the smooth deformation of neighboring nodes:

(4) $$E_{s}\left(A,T\right)=\sum_{(i,j)\in \mu }\left|\right|A_{i}\left(g_{j}-g_{i}\right)+g_{i}+t_{i}-\left(g_{j}+t_{j}\right){\left|\right|}_{2}^{2}.$$

Finally, the critical part will be how to collect correspondences between the source and target mesh. In this paper, we have incorporated correspondences extracted from both geometric cues and color cues.

For the geometric term $E_{g}$, the correspondences between $M_{k-1}$ and $M_{k}$ are established via the deformed mesh $S_{k-1}$ that we have recorded during the partial piece fusion procedure in Section 4.1.1. The deformed mesh $S_{k-1}$ is supposed to have roughly good initial alignment with $M_{k}$, since the mesh $S_{k-1}$ has actually been optimized to confront with the last frame of sequence k−1 from the canonical mesh $M_{k-1}$ and this last frame of segment k−1 is just the first frame for *k*th segment. Therefore, we can establish the correspondences between $S_{k-1}$ and $M_{k}$ using nearest search. After that, those correspondences will be transferred from $S_{k-1}$ to $M_{k-1}$, given that the corresponding vertices of $M_{k-1}$ and $S_{k-1}$ share the same vertices indexes. This will make the alignment between neighboring segments much easier to achieve. The correspondences are updated after several iterations during the optimization in an ICP manner. We define this term in Equation (5) with $C_{g}$ denotes the correspondences set:

(5) $$E_{g}\left(A,T\right)=\sum_{\left(p_{i},q_{i}\right)\in C_{g}}||\phi \left(p_{i}\right)-q_{i}{\left|\right|}_{2}^{2}.$$

**Figure 4:**
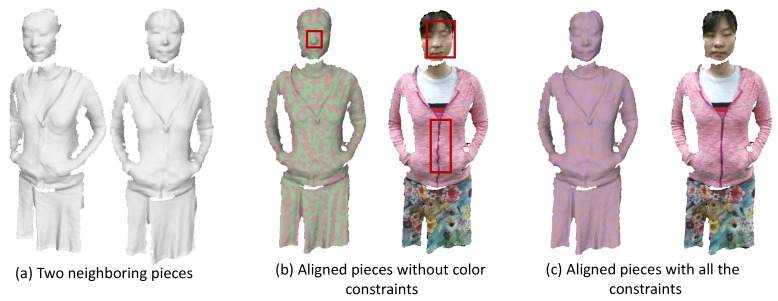
Illustration of pairwise alignment results. (**a**) two neighboring pieces achieved from the above partial piece generation step; (**b**) the alignment results without the color information. These two pieces are shown with different colors in the left of (**b**) so that we can see the alignment result more clearly. The misalignment in the appearance is visualized in the right figure of (**b**) where the two meshes colored by the captured color images are overlaid; (**c**) demonstrates the alignment result with all those constraints in Equation (2) where we can see that the two meshes are well-aligned.

However, as the nearest searching strategy is not guaranteed to provide the correct matches (as shown in Figure 4b), we also exploit the color information to resolve the ambiguity. Specifically, we need to compute the dense 3D flow from the colored mesh $S_{k-1}$ to $M_{k}$. For the classical scene flow computation from two input RGB-D images, we use the two-dimensional image plane as the parameterization domain to optimize the flow field. However, in our case, the unstructured point clouds do not provide a natural parametrization domain.

**Figure 5:**
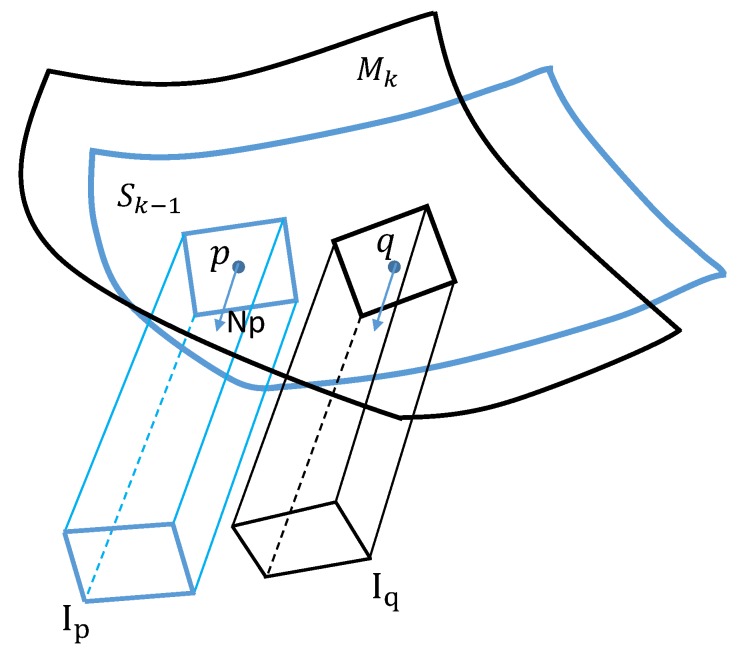
Illustration of the virtual plane to define color matches. p is a vertice on mesh $S_{k-1}$ with its normal $n_{p}$. q is the nearest vertice to p on mesh $M_{k}$. The neighboring verices around p and q are projected under the direction of $n_{p}$ to get the rendered image $I_{p}$ and $I_{q}$, respectively. The neighboring might not form a rectangular, and we just use a rectangle box for simplification of illustration.

We address this problem by defining a virtual image on the tangent plane of every point and projecting the colored vertices around that point onto the virtual plane. In more detail, for every vertice p in mesh $S_{k-1}$, we gather its *K* nearest connected faces around p and render this neighboring colored mesh piece orthogonally onto the plane $Pl_{p}$ defined by the vertice p and its normal $n_{p}$. The rendered image patch $I_{p}$ can be seen as a local approximation of the colored mesh around vertice p. We parametrize the colored mesh locally by the virtual plane. For the vertice p, the corresponding nearest vertice in mesh $M_{k}$ has been computed from the geometric term, and we denote it as q. Similarly, we gather the faces around q and, by rendering this mesh fragment onto the plane $Pl_{p},$ we get the rendered image patch as $I_{q}$. The procedure is illustrated in Figure 5. The dense matches between $I_{p}$ and $I_{q}$ are found by the calculation of the flow field between these two image patches followed by a cross check validation step to filter out outliers. Those matches provide us correspondences between mesh $S_{k-1}$ and $M_{k}$ as we keep track of the mapping from 3D vertices to the rendered 2D image patches.

Another issue that will arise in the above procedure is that for some vertice p in mesh $S_{k-1}$, more than one correspondence may be found in mesh $M_{k}$ since it might be collected by multiple vertices as neighbors. Therefore, we set the correspondence as the median of the multiple corresponding vertices to reduce the affect of outliers. Finally, we get the correspondence set $C_{c}$ between these two colored meshes after the above process and arrive at the color energy term defined as follows:

(6) $$E_{c}\left(A,T\right)=\sum_{\left(p_{i},q_{i}\right)\in C_{c}}||\phi \left(p_{i}\right)-q_{i}{\left|\right|}_{2}^{2}.$$

We demonstrate the effectiveness of the color correspondences matching term in Figure 4.

By now, with all the constraints defined, we can minimize the objective function of Equation (2) to get the unknown deformation parameters A and T. This optimization problem can be solved with the Levenberg–Marquardt algorithm. Afterwards, we can apply the deformation field to all the vertices in $M_{k-1}$ via Equation (1) and we will get the deformed mesh $T_{k-1}$ that is aligned with $M_{k}$.

### 4.2. Loop Detection and Global Non-Rigid Registration

**Figure 6:**
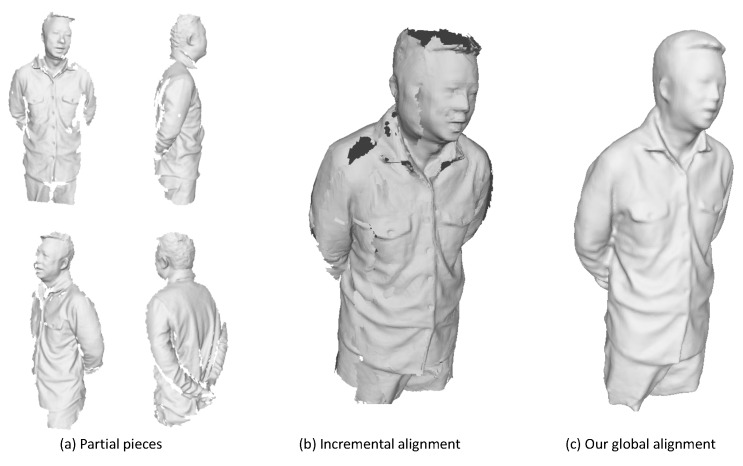
Illustration of drifting effect of incremental alignment and comparison with the result after our global registration. (**a**) some sampled partial pieces; (**b**) the result from incremental alignment where large gaps exist; (**c**) the result after global registration.

After the above pairwise alignments of meshes from neighboring segments, we are ready to find reliable correspondences between neighboring pieces. We can certainly align those canonical models incrementally into the first piece using techniques as described in Section 4.1.2. However, the drifting problem is almost inevitable during the sequential alignment. As shown in Figure 6, the large gaps between the first and last pieces stops the sequential alignment strategy from getting complete and visually plausible models. We argue that the two key aspects of assembling those pieces are loop detection and global non-rigid registration. We describe these two procedures in the following sections.

#### 4.2.1. Loop Detection

In our case, loop detection is to find the partial pieces that have sufficiently large overlap with the first piece, that is, while the subject rotates in front of the sensor, we want to find the piece where he/she has rotated all around and arrived back to the first frame. In this section, we develop the loop detection strategy exploiting those SIFT features as similar to the loop detection in the SLAM system [3], where the Bag of Words has often been used. During the partial pieces generation procedure (Section 4.1.1), the SIFT features have been extracted and matched to assist the tracking [17]. For each frame, the matched features are lifted and stored in the 3D canonical space. Therefore, for each mesh $M_{k}$, we have some sparse features associated with it.

Our goal will be to find some pieces among $M_{2}∼M_{N}$ that have great overlap with $M_{1}$ given those canonical models $M_{1}∼M_{N}$ with sparse SIFT feature descriptors attached.

First, for each model $M_{k}$ (k=2 to *N*), we find the matches of SIFT features within certain matching threshold between mesh $M_{1}$ and $M_{k}$. Then, we evaluate the degree of coverage of those matches with respect to the surface. It is assumed that, if the matches reside only on a small part of the model, it implies that these two models do not have sufficient overlap. Otherwise, these matches would spread over the surface. However, it is still not sufficient to simply use this to define the extent of overlap, as the SIFT vertices might scatter over the surface unevenly, which will also cause the uneven distribution of overlap over the surface.

Thus, taking both factors into consideration, we evaluate the coverage adaptively on different regions depending on the distribution of the SIFT vertices. First, we sample a set of vertices (P) over the surface and we compute the coverage degree of SIFT features around each sampled vertice. The coverage degree $f_{s}$ of SIFT features for each vertice v is measured via $f_{s}\left(v\right)=exp\left(-d_{s}{\left(v\right)}^{2}/\sigma_{ds}^{2}\right),$ where $d_{s}\left(v\right)$ is the distance to the nearest feature on the mesh. Similarly, the coverage degree of matches $f_{m}\left(v\right)$ is computed with $f_{m}\left(v\right)=exp\left(-d_{m}{\left(v\right)}^{2}/\sigma_{dm}^{2}\right),$ where $d_{m}\left(v\right)$ is the distance to the nearest match. The coverage score is defined as $S=1/\left|P\right|\sum_{v\in P}\left[f_{m}\left(v\right)/f_{s}\left(v\right)\right]$. The larger the score, the larger the coverage of matches. Ideally, if the two meshes are identical, the score should be equal to 1. Therefore, we select *L* (L=2) pieces from $M_{2}∼M_{k}$ that have the largest coverage score with respect to the mesh $M_{1}$.

#### 4.2.2. Global Non-Rigid Registration

In this section, we present how to enforce those loop constraints to achieve a global registration. Similar to the pairwise registration part, the Embedded Deformation Model is also employed here to extrapolate the deformation field. First, we build up and embed a deformation graph for every piece of mesh ($M_{1}$ to $M_{N}$). Our goal will be to optimize the deformation parameters ($A=A_{1}∼A_{N}$, $T=T_{1}∼T_{N}$) of all those graphs altogether. For each $A_{i}$ and $T_{i}$, they are a set of affine matrices and translation vectors, respectively, which are associated with the deformation graph embedded in mesh $M_{i}$. Following the technique in [28], the objective function is formulated as

(7) $$E\left(A,T\right)=\sum_{i=1}^{N}\left[\alpha_{rigid}E_{r}\left(A_{i},T_{i}\right)+\alpha_{smooth}E_{s}\left(A_{i},T_{i}\right)\right]+\alpha_{corr}E_{corr}.$$

The first two terms in the above equation are the as-rigid-as-possible term and smooth term, respectively, as defined in Equations (3) and (4). We have the third term $E_{corr}$ enforcing the correspondences’ constraints, which is the most critical part. In our case, we have two sets of correspondences including the those established between neighboring pieces ($E_{corr\_nei}$) and those from pieces that form a loop ($E_{corr\_loop}$):

(8) $$\begin{array}{c} E_{corr}=E_{corr\_nei}+E_{corr\_loop}=\sum_{k=1}^{N-1}\sum_{\left(p_{i},q_{i}\right)\in C_{k}}||\phi \left(M_{k}^{p_{i}},A_{k},T_{k}\right)-M_{k+1}^{q_{i}}{\left|\right|}_{2}^{2}\\ +\sum_{k\in Lp(1)}\sum_{\left(p_{i},q_{i}\right)\in C_{k}}||\phi \left(M_{k}^{p_{i}},A_{k},T_{k}\right)-M_{1}^{q_{i}}{\left|\right|}_{2}^{2}. \end{array}$$

For the first part, it incorporates the constraints between neighboring pieces $M_{k}$ and $M_{k+1}$. After the pairwise registration from Section 4.1.2, we are ready to find correspondences of neighboring pieces by the nearest search since those pieces have already been aligned. In practice, we do not need to enforce all those matches; instead, we randomly sample about 300–400 correspondences for every two pieces. We denote the correspondence set between $M_{k}$ and $M_{k+1}$ as $C_{k}$.

Second, for those pairs of pieces that have been marked as a loop, the correspondences between them play an important role in global registration by enforcing the loop closure constraints ($E_{corr\_loop}$). The key problem now is how to register those pairs of pieces to get the correspondences.

Finding reliable matches between those pairs of pieces is not a trivial problem due to the more complicated non-rigid deformation and also the drifting issue. The real correspondences might have quite a large distance, which makes the nearest searching strategy not proper in this case. Therefore, we cannot simply apply the pairwise registration algorithm proposed in Section 4.1.2. Another possible solution would be to adopt the sparse SIFT features to match correspondences. However, the problem is that there might not be features extracted in textureless regions, and to make things even worse, we cannot guarantee those matches to be reliable. To deal with this issue, we propose here to exploit the dense flow information of those two colored meshes.

Now, suppose we want to align the colored mesh $M_{c}$ to the first piece $M_{1}$. First, we apply rigid registration between those two meshes to make them roughly aligned. Afterwards, we generate two color images $I_{c}$ and $I_{1}$ by rendering $M_{c}$ and $M_{1}$, respectively, under the camera projection of mesh $M_{1}$. Instead of searching correspondences locally as in the pairwise registration approach, we compute the dense optical flow globally from the rendered image $I_{c}$ to $I_{1}$. Considering that the flow displacement might be quite large under the non-rigid deformation, we exploit the method from paper [29] to adopt HOG features into the flow computation framework to handle large displacement flow.

Next, to validate those matches and remove outliers, we exploit the 3D geometry information of the two meshes. The intuitive way is to reject those candidate matches for which the distance is quite large in 3D space. However, given that the subject is experiencing non-rigid deformation, we cannot be sure how large the deformation would be. We might actually remove some potential true correspondences if we set the threshold of the distance to be small; on the other hand, outliers might not be filtered out if we set it to be large. To handle this issue, we propose a more intelligent filtering strategy under an as-rigid-as-possible principle.

Now, suppose we have a pixel *p* in $I_{c}$ that has its corresponding pixel *q* in $I_{1}$, which has been acquired from the computed flow field. For pixel *p*, we have its corresponding vertice on mesh $M_{c}$ denoted as $v_{p}$. Its neighboring vertices $N_{v}$ within some certain distance on the mesh can be extracted. In addition, we make use of the geodesic distance here to keep the extracted neighboring vertices to be connected. The corresponding pixels for those vertices $N_{v}$ on the rendered image $I_{c}$ are denoted as $N_{p}$. With the computed flow field, we can obtain the correspondences of $N_{v}$ on the mesh $M_{1}$. Those corresponding vertices are denoted as $N_{v}^{\prime }$.

From the corresponding vertices set $N_{v}$ and $N_{v}^{\prime }$, we approximate the rigid transformation $R_{v}$, $T_{v}$ ($R_{v}$ is a 3×3 rotation matrix and $T_{v}$ is a 3×1 translation vector) by minimizing the following energy function:

(9) $$E\left(R_{v},T_{v}\right)=\sum_{i=1}^{|N_{v}|}\left|\right|\left(R_{v}N_{v}^{i}+T_{v}\right)-{N_{v}}^{{}^{\prime }i}{\left|\right|}_{2}^{2}.$$

To eliminate the affect of outliers, we adopt a RANSAC procedure to find the best rigid transformation that will align those two vertice sets. Afterwards, under the assumption of locally as-rigid-as-possible deformation, if the deformation of vertice $v_{p}$ confronts the estimated transformation $R_{v}$, $T_{v}$, we would say that this is potentially a good match. Otherwise, the match we get from the flow field for pixel *p* will be regarded as an outlier. We measure the deformation consistency using the following equation:

(10) $$M_{d}=exp\left(-\frac{\left|\right|\left(R_{v}v_{p}+T_{v}\right)-v_{p}^{\prime }{\left|\right|}_{2}^{2}}{2\sigma_{M}^{2}}\right).$$

We remove matches with $M_{d}$ smaller than a threshold.

To this point, all the constraints in Equation (7) have been built up and we are ready to solve the optimization problem to get the optimal deformation parameters that will align all those pieces together and form a complete 3D model. The results after this registration are shown in Figure 6c. Deformed meshes after the global non-rigid registration are represented as $M_{1}^{g}∼M_{N}^{g}$.

**Figure 7:**
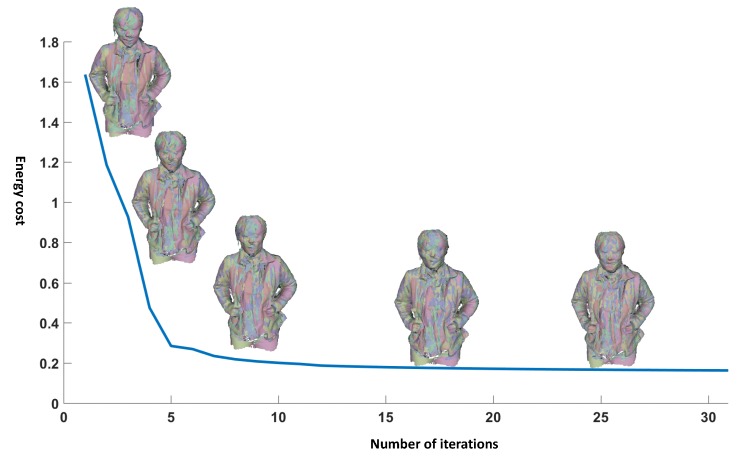
Illustration of the global registration evolution process.

We demonstrate the evolution process for the global registration optimization in Figure 7 showing the curve of the energy cost with respect to number of iterations. The optimization gets converged after a few iterations.

### 4.3. Model Update

**Figure 8:**
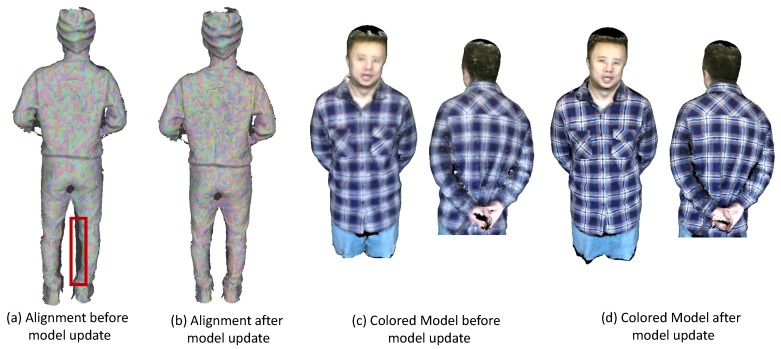
Illustration of results before and after model update. (**a**,**b**) show all the aligned pieces before and after the model update step, respectively. The misalignment still exists around the legs as marked red after the global registration. The alignment has gotten better after our model update as shown in (**b**). Some colored models are shown in (**c**,**d**). The appearance before model update (**c**) is quite blurry, while more clear and consistent color maps have been achieved after the model update (**d**).

At this point, we have got a fairly good 3D model of the deformable object, whereas there are still some artifacts caused by misalignment as shown in Figure 8a,c. We found out that the major reason for the misalignment is surface occlusion. Specifically, if some part of the subject has been captured and modeled in piece $M_{k}$, which is then being occluded in the next piece $M_{k+1}$, the part reappears in piece $M_{k+2}$. Then, misalignment might show up between $M_{k+1}$ and $M_{k+2}$ in the overlapping region since we haven’t enforced any constraints explicitly between these two pieces during the previous alignment procedure. One simple and naïve way to handle this would be to apply non-rigid pairwise alignment between $M_{k}$ and $M_{k+2}$ to establish reliable correspondences. However, first, we wouldn’t know when this kind of misalignment will occur and, second, the overlap between $M_{k}$ and $M_{k+2}$ might be fairly small, which makes it even harder to find reliable correspondences.

Therefore, to deal with this kind of misalignment, we take advantage of the current model (denoted as $V_{0}$) that we have reconstructed after the global non-rigid alignment step and take it as a starting point to update and refine the model. Essentially, we want to find the optimal model that will confront all those pieces both in its geometry and appearance. Instead of exploiting the expensive bundle adjustment strategy, we intend to update the model iteratively by deforming those pieces onto this proxy model. Algorithm 1 shows our procedure for updating the model.

First of all, at the initialization step, we deform the first piece $M_{1}^{g}$ to the current proxy model $V_{0}$, which is a trivial problem since $V_{0}$ is recovered with the first piece as the canonical frame. Afterwards, we attach the vertices color to the proxy model from the region covered by $M_{1}^{g}$ via nearest search. That is, for each vertice *v* in $V_{0}$, we find its nearest vertice $v_{m}$ in $M_{1}^{g}$ and set the color of *v* to be same as $M_{1}$ if $|v-v_{m}|<Thres$. After this initialization step, we get the proxy model that is partially colored.

For step 2, we update the proxy model $V_{0}$ with respect to each of those pieces. $V_{0}$ covers the whole model while each piece $M_{k}^{g}$ only covers part of the model. Therefore, instead of deforming $V_{0}$ to align with the mesh $M_{k}^{g}$ (*k* starts from 2 to *N*), we align those meshes towards the current model $V_{0}$ exploiting the method proposed in Section 4.1.2 that utilizes both geometric and color information to achieve better alignment. We denote the deformed mesh of $M_{k}^{g}$ as $M_{k}^{g^{\prime }}$.

Then, correspondences between the mesh $M_{k}^{g}$ and the proxy model are established via nearest search between $M_{k}^{g^{\prime }}$ and $V_{0}$. We deform the geometry of the proxy model under the guidance of those correspondences with Laplacian constraints. In the meantime, the vertices color in $M_{k}^{g}$ can be transferred to the proxy model as described in the initialization step. In addition, we update the appearance (the vertex color) of the proxy model as the weighted average of the current vertices color and the vertices color acquired from $M_{k}^{g}$.

For step 3, after finishing the iteration for each piece of the segment in step 2, we re-apply the global non-rigid registration for the pieces $M_{k}^{g}$ (*k* from 1 to *N*). The correspondence term in Equation (7) is built up by nearest search between every two pieces of the deformed meshes $M_{1}^{g^{\prime }}$– $M_{N}^{g^{\prime }}$.

We iteratively go over the above steps for better alignment and updating of the model. In addition, we will finally arrive at our reconstructed colored model that has good quality in both geometry and appearance as shown in Figure 8d.

**Algorithm 1:** Model-update algorithm.
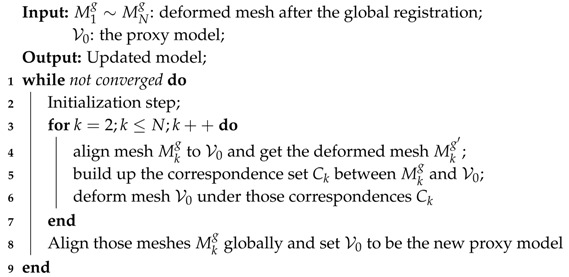


### 4.4. Implementation Details

The overall pipeline is performed offline while the partial pieces generation part can be done in real time [17]. It takes about 40 min overall to run in Matlab 2016a on a desktop with 8-core 3.6 G Hz Intel CPU and 16 GB memory. In more detail, the pairwise registration part takes about 5 min and around 20 min are taken in global registration part. The final model update part takes about 15 min. The loop detection part takes little time as compared to those registration procedures.

The parameters used in the paper are set with $\alpha_{r}=100,\alpha_{s}=1000,\alpha_{g}=1.0,\alpha_{c}=1.0,\alpha_{rigid}=50,\alpha_{smooth}=500,\alpha_{corr}=1.0$.

## 5. Experiments

We demonstrate the effectiveness of our approach in the experimental part with both quantitative and qualitative results. Furthermore, we present an application of model completion using our recovered 3D model.

### 5.1. Quantitative Evaluation on Rigid Objects

**Figure 9:**
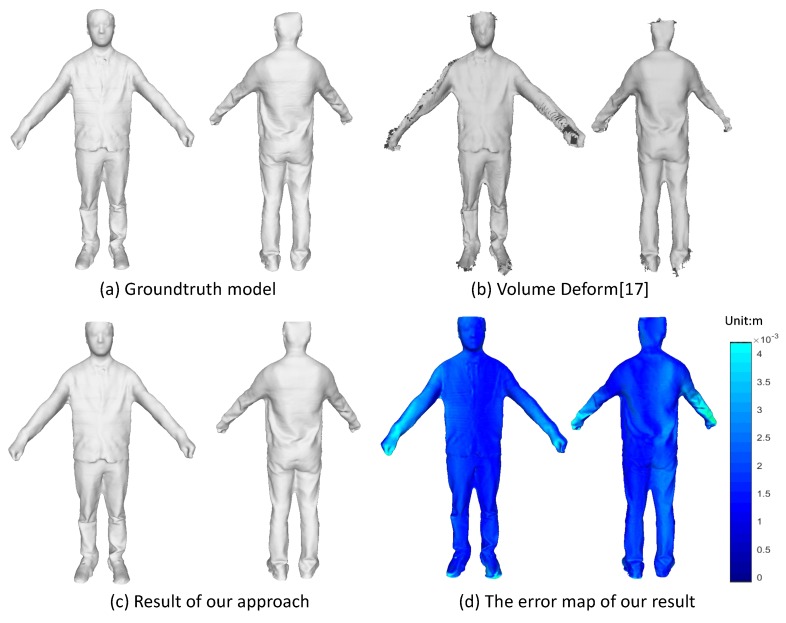
Quantitative evaluation on synthetic dataset.

Even though we target on the non-rigidly deformable objects, it does not stop us from implementing our approach on the rigid objects. It is more convenient to take advantage of the rigid objects for quantitative evaluation. Here, we use a textured mesh model scanned by a multi-view scanner system as the groundtruth and synthesize a sequence of depth and color images by moving a virtual camera around the 3D mesh model. We run both the VolumeDeform [17] and our method on this synthetic data with the results shown in Figure 9. We plot the error map to show the geometric error of our reconstructed model as compared with the groundtruth model. The error for each vertice is computed via a nearest search from this vertice to the groundtruth mesh model. As we can see from the 3D error map in Figure 9c, the most largest error (about 0.0041 m) comes from the part of arms and hands, which have relative thin structure and are more difficult to track and align. From the overall model, we get the mean error as 0.0023 m. The result demonstrates that we can get a recovered model that is fairly accurate.

### 5.2. Qualitative Evaluation on Captured Subjects

For the qualitative evaluation, we have captured several sequences of human subjects with Microsoft Kinect V2. The human subject is asked to rotate in front of the Kinect sensor.

**Figure 10:**
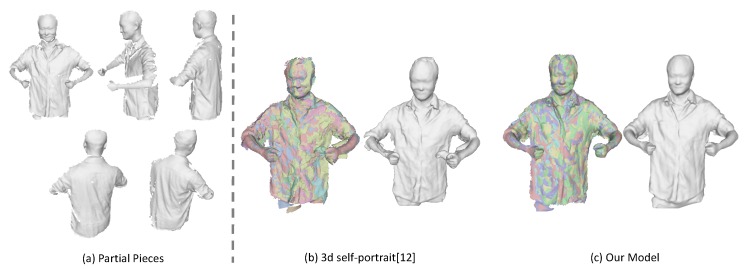
Comparison with 3D self-portrait. (**a**) some sampled partial pieces; (**b**) the results from 3D self-portrait [12]; (**c**) the results we get with our approach. We have colored the deformed pieces with different colors to better demonstrate the alignment results.

First, we compare our results with a 3D self-portrait [12], which takes eight partial pieces as input. We run the method on one of our captured sequences for which the non-rigid motion is minimal among all the sequences and the subject has tried to stay at the same pose during rotation. We have manually selected eight frames from the sequence that evenly distributed across a cycle. The comparison results are shown in Figure 10. As the almost inevitable non-rigid motion problem during rotation, the misalignment still exists for the 3D self-portrait method especially around the arms, which can be seen in Figure 10b. On the contrary, we are able to align those partial pieces successfully under our framework, as we have kept tracking the non-rigid motion continuously. The results of our method is displayed in Figure 10c.

**Figure 11:**
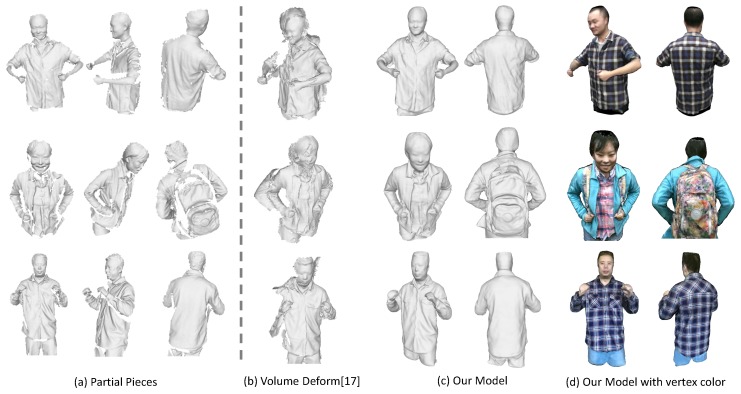
Qualitative evaluation on upper body models. (**a**) some sampled partial pieces; (**b**) the results from VolumeDeform [17]; (**c**) the complete models we get with our approach; (**d**) our recovered colored models.

**Figure 12:**
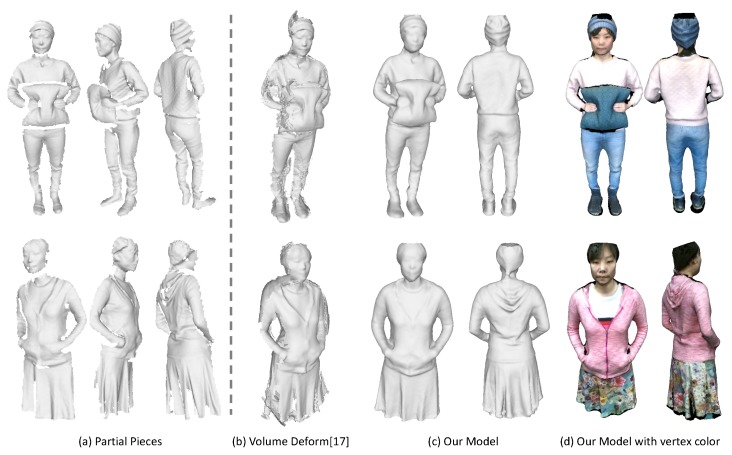
Qualitative evaluation on full body models. (**a**) some sampled partial pieces; (**b**) the results from VolumeDeform [17]; (**c**) the complete models we get with our approach; (**d**) our recovered colored models.

To compare with previous dynamic fusion methods, we implement a sequential dynamic fusion method [17] that fuse the frames incrementally but without concerning the loop closure. Figure 11 shows the comparison results of the upper body of some human subjects. Figure 12 presents some results on the full body modeling, which is more challenging considering the inevitable occlusion and large deformation for the legs. As compared to the method [17], which shows large gaps in the recovered model, we are able to get a complete and watertight model since we have enforced the loop closure constraints explicitly to solve error accumulation problem. Although we haven’t achieved real-time performance, we can get much better results as compared with the dynamic fusion methods. In addition, since we haven’t enforced any constraints on the subjects, we are also able to deal with more general cases where the human subject is holding something or carrying a backpack. We can also reconstruct the girl in a shirt, which has experienced free-form deformation as she moves.

As shown in these figures, the recovered color maps of those models are quite clear and edges are sharp. We can see the textures on the surface clearly. This is achieved by our registration method that has incorporated both geometric and appearance constraints. The parts that haven’t been observed (e.g., under the chin or inner side of the arm) are colored as black. This could be filled up by color of neighboring vertices, while we haven’t put our effort in this.

### 5.3. Applications

**Figure 13:**
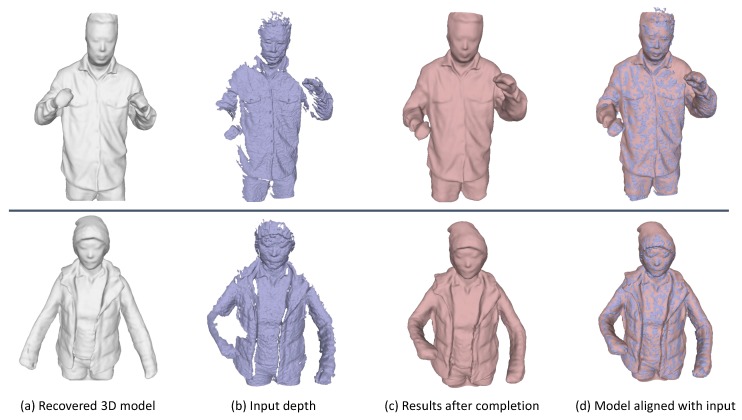
Applications on model completion. (**a**) the recovered 3D models in canonical space from our proposed framework; (**b**) some input depth frames which capture only partial of the model; (**c**) the results after model completion; (**d**) the aligned meshes of our model after completion and the input meshes.

Given the complete 3D model that we have recovered from our proposed framework, we are able to drive or deform the model for some model completion applications. That is, given a depth frame as input that has quite limited coverage of the model, we can perform the completion by deforming the 3D model that we have got to get it aligned with the current input. We have employed the registration technique that we have proposed in Section 4.1.2 to accomplish this task. Some completion results are shown in Figure 13.

## 6. Conclusions

In this paper, we have proposed a framework to reconstruct the 3D shape and appearance of the deformable objects under the dynamic scenario. To tackle the drifting problem during the sequential fusion, we have partitioned the entire sequence into several segments, from which we have reconstructed partial scans. A global non-rigid registration approach is applied to align all those pieces together into a consistent canonical space. We achieve this with our loop closure constraints to help eliminate the accumulation error. Afterwards, the recovered model gets updated with our novel model update method to arrive at our final model with accurate geometry and high fidelity of color maps. During the non-rigid alignment and loop closure procedure, we have exploited both geometric and appearance information to resolve the ambiguity of matching. The framework has been validated on both synthetic and real datasets. We are able to recover 3D models with accuracy in millimeters as demonstrated from our quantitative evaluation. Experiments on real datasets demonstrate the capability of our framework to reconstruct complete and watertight deformable objects with high fidelity color maps.

Looking into the future, we would like to further improve our method by replacing the per vertex color representation of the mesh with textures to get even higher quality of mesh appearance. The changing topology could be another direction that we will investigate as for now the topology is restricted to be constant throughout the sequence. In addition, our method relies on the success of building up partial scans, which might fail in case of fast motion. We believe that it could be solved by adopting the learning based approaches to find correspondences instead of using nearest search or projective association. Various applications (e.g., model based view synthesis) could be developed based on our work.
